# Reliability of the Transmission Line Method and Reproducibility of the Measured Contact Resistance of Organic Thin-Film Transistors

**DOI:** 10.1021/acsnano.4c15828

**Published:** 2025-03-10

**Authors:** Tobias Wollandt, Sabrina Steffens, Yurii Radiev, Florian Letzkus, Joachim N. Burghartz, Gregor Witte, Hagen Klauk

**Affiliations:** † Max Planck Institute for Solid State Research, Heisenbergstr. 1, Stuttgart 70569, Germany; ‡ Molecular Solids Group, Philipps-Universität Marburg, Renthof 7, Marburg 35032, Germany; § Institute for Microelectronics Stuttgart (IMS CHIPS), Allmandring 30A, Stuttgart 70569, Germany

**Keywords:** organic TFTs, contact resistance, TLM, statistics, environmental parameters

## Abstract

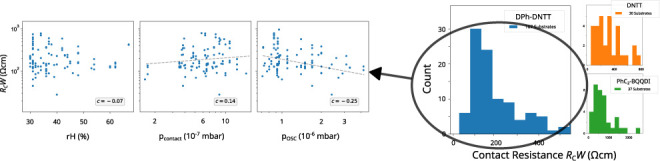

Using the transmission line method (TLM), we extracted the contact resistance of organic thin-film transistors (TFTs) based on five different vacuum-deposited small-molecule semiconductors fabricated on over 500 substrates. In the first part of this report, we illustrate how the reliability of the TLM analysis is affected by the statistical uncertainty that arises from the fitting procedure and by the systematic error that is introduced if the actual channel length of the TFTs deviates from the nominal channel length. In the second part, we show that the contact resistance of organic TFTs varies significantly from one fabrication run to the next (and even across substrates fabricated within the same fabrication run), no matter how much care is taken to keep all controllable fabrication-process parameters constant. A statistical analysis reveals no strong correlations between the contact resistance and environmental parameters present during TFT fabrication, such as the humidity in the laboratory or the base pressure of the vacuum during material depositions. This suggests that the observed variation in the contact resistance is mainly stochastic. For the TFTs based on the best-performing semiconductor, the contact resistance varies between 28 Ωcm and 1 kΩcm, with a median value of 160 Ωcm.

The dynamic performance of organic thin-film transistors (TFTs) depends on a variety of device parameters such as the charge-carrier mobility, the channel length, the gate-dielectric capacitance, and the contact resistance. However, it has been shown that when the channel length is below about 10 μm and the intrinsic channel mobility is greater than about 1 cm^2^/(V s) (which is the case for most high-performing organic TFTs reported in literature), then the transit frequency of organic TFTs will be limited mainly by the contact resistance.^1^ A better understanding of what determines the contact resistance of organic TFTs and how it can be further reduced is thus of great importance.^2−4^

The channel-width-normalized contact resistance of organic TFTs reported in the literature (more than 400 reports) varies from about 10^9^ Ωcm to as small as 1 Ωcm.^5^ Such a wide range (9 orders of magnitude) can be explained mainly by the fact that organic TFTs are being fabricated using a wide range of functional materials, fabrication methods, and device architectures. For example, for TFTs fabricated in the bottom-gate, top-contact (inverted staggered) device architecture, Kraft et al.^6−8^ and Rolin et al.^9^ reported contact resistances ranging from 0.16 to 13.8 kΩcm, depending on the choice of the organic semiconductor (DPh-DNTT, DNTT, C_10_-DNTT, C_10_-DNBDT, C_8_-BTBT, tetracenothiophene, anthradithiophene, pentacene). For C_10_-DNTT TFTs fabricated in the bottom-gate, top-contact device architecture, Zeng et al.^10^ reported contact resistances ranging from 14 to 800 Ωcm, depending on the contact metal (Pt, Au) and the metal-deposition process (vacuum deposition, lamination). For poly(3-hexylthiophene) TFTs fabricated in the bottom-gate, bottom-contact (inverted coplanar) device architecture, Bürgi et al.^11^ reported an even stronger dependence on the contact metal (Au, Ag, Cu, Cr), with contact resistances ranging from 5 kΩcm to 5 MΩcm. For TFTs fabricated in the staggered top-gate device architecture using TIPS-pentacene as the semiconductor and Au for the source/drain contacts, Choi et al.^12^ reported contact resistances ranging from 11 to 351 kΩcm, depending on the contact treatment (MoO_3_, Mo(tfd)_3_, PFBT). For bottom-gate DPh-DNTT TFTs, Borchert et al.^13^ reported contact resistances of 29 Ωcm for TFTs fabricated in the coplanar device architecture and 56 Ωcm for TFTs fabricated in the staggered device architecture. These results illustrate how important the details of the fabrication process are for device performance, especially for the contact resistance.

However, even when organic TFTs are fabricated using the same device architecture and identical materials and methods, the measured contact resistance often varies noticeably between individual fabrication runs. This obviously renders the development of reliable approaches to the fabrication of organic TFTs that reproducibly exhibit low contact resistance quite challenging. In the pursuit of a better understanding of such variability, we report on a study in which we fabricated several hundred substrates with organic TFTs over a period of several years and extracted the contact resistance of the TFTs using the transmission line method (TLM) within two hours after device fabrication. The measured contact resistances are close to the smallest values reported for the respective semiconductors. However, we found an unexpectedly large variation in the measured contact resistance despite the fact that identical materials and methods were employed for the fabrication of the TFTs. Here, we focus mainly on TFTs fabricated in the bottom-gate, bottom-contact (inverted coplanar) device architecture (illustrated in Figure 1a), using the vacuum-deposited small-molecule semiconductor 2,9-diphenyl-dinaphtho[2,3-*b*:2',3′-*f*]thieno[3,2-*b*]thiophene (DPh-DNTT) (molecular structure shown in Figure 1b).^14^ The source/drain contacts were prepared using vacuum-deposited gold, functionalized with a chemisorbed monolayer of pentafluorobenzenethiol (PFBT) to increase its work function.^15^ This combination of device architecture and functional materials was chosen since TFTs fabricated in this manner were previously found to have the smallest contact resistance reported so far for organic TFTs (10 Ωcm),^16,17^ aside from two reports of electrolyte-gated organic TFTs whose contact resistance benefits from the large carrier density induced in the semiconductor by the electrolyte.^5,18^ For the coplanar DPh-DNTT TFTs considered here, we measured contact resistances ranging from 28 Ωcm to almost 1 kΩcm. To illustrate that this large variation of the contact resistance is not unique to this particular device architecture or this particular combination of materials, we additionally show results from TFTs fabricated in the bottom-gate, top-contact (inverted staggered) device architecture and from TFTs fabricated by using other organic semiconductors. The relevant energy levels of these materials are summarized in Figure S1.

**Figure 1:**
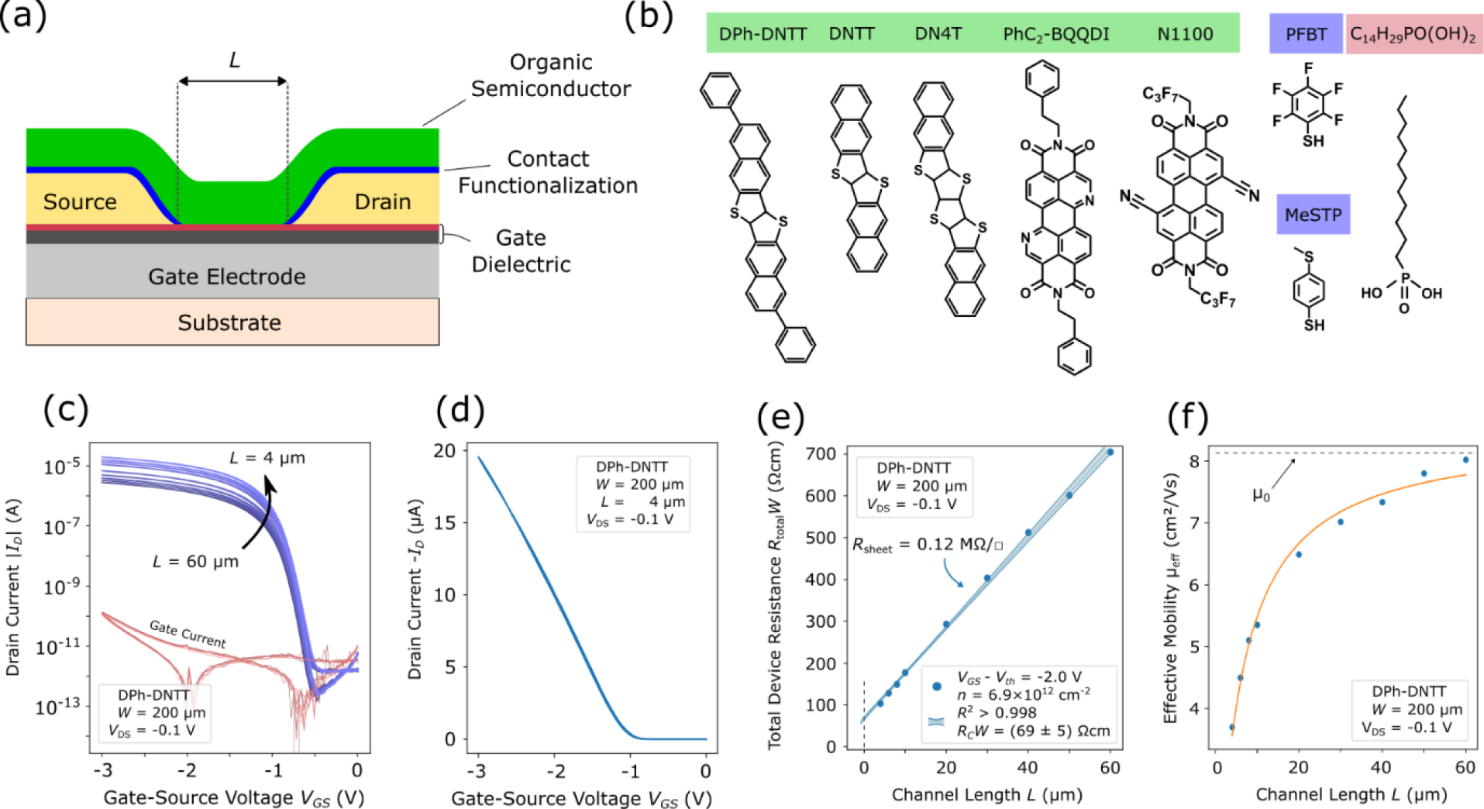
(a) Schematic cross-section of organic TFTs fabricated in the bottom-gate, bottom-contact (inverted coplanar) device architecture. (b) Chemical structures of the molecules used in this study: organic semiconductors DPh-DNTT, DNTT, DN4T, PhC_2_–BQQDI and N1100, thiols PFBT and MeSTP for contact functionalization, and *n*-tetradecylphosphonic acid as part of the hybrid gate dielectric (having a unit-area capacitance *C*_diel_ of 0.6 μF/cm^2^). (c) Typical transfer characteristics of DPh-DNTT TFTs with channel lengths ranging from 4 to 60 μm, measured at a drain-source voltage of −0.1 V. (d) Transfer characteristic of a DPh-DNTT TFT with a channel length of 4 μm. (e) TLM analysis: total device resistance (*R*_total_ = *V*_DS_/*I*_D_) at a gate-overdrive voltage (*V*_GS_ – *V*_th_) of −2 V (corresponding to a charge-carrier concentration of 6.9 × 10^12^ cm^–2^) plotted versus the channel length. The statistical fitting error (uncertainty) σ is indicated by the confidence interval. (f) Effective charge-carrier mobility μ_eff_ plotted vs the channel length. From this graph, the intrinsic channel mobility μ_0_ can be extracted by fitting eq 4 to the data.

To try to explain the large variation in contact resistance observed in this study, we investigated possible correlations between the measured contact resistances and the environmental parameters that were present at the time when the TFTs were fabricated and characterized. These parameters include the ambient humidity in the laboratory and the base pressure in the vacuum system during the deposition of the organic semiconductor and the source/drain metal. The correlations we found between these environmental parameters and the measured contact resistance are weaker than anticipated; even the largest correlation coefficient is no greater than *c* = 0.25.

## Results and Discussion

Given the importance of extracting the contact resistance properly, we will start by briefly reviewing some of the considerations when applying the TLM, in particular, the importance of accurately determining the actual channel length of the TFTs and the influence of the sheet resistance of the semiconductor.

### General Approach

The transmission line method (TLM) separates the total device resistance *R*_total_ into the channel resistance *R*_ch_ (associated with charge transport through the semiconductor in the lateral direction) and the contact resistance *R*_C_ (the sum of source resistance *R*_C,S_ and drain resistance *R*_C,D_), as shown in eq 1. The contact resistance is obtained by extrapolating the linear fit of the total device resistance as a function of channel length *L* to a channel length of zero, where *R*_total_ = *R*_C_, as seen from eq 2. To allow benchmarking, the resistances are normalized to the channel width, i.e., *R*_total_*W* and *R*_C_*W.*
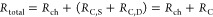

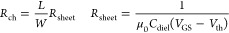
1
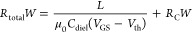
2

Here, *R*_sheet_ is the sheet resistance of the semiconductor layer, μ_0_ is the intrinsic channel mobility, *C*_diel_ is the unit-area capacitance of the gate dielectric, *V*_GS_ is the gate-source voltage, and *V*_th_ is the threshold voltage. Note that channel resistance *R*_ch_ depends on *L*, while contact resistance *R*_C_ is independent of *L*. The total device resistance *R*_total_ is obtained by measuring the drain current *I*_D_ as a function of the applied gate-source voltage *V*_GS_ for a fixed drain-source voltage *V*_DS_, so that *R*_total_ = *V*_DS_/*I*_D_. Within the gradual channel approximation and in the linear regime of operation (*V*_DS_ ≪ *V*_GS_ – *V*_th_), the drain current is described by
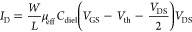
3where μ_eff_ is the effective charge-carrier mobility.

Figure 1c–e illustrates the extraction of the device parameters. For each channel length, the linear increase of *I*_D_ in the transfer characteristic is fitted with eq 3 to extract the threshold voltage *V*_th_ and the effective charge-carrier mobility μ_eff_. The intrinsic channel mobility μ_0_ can be either extracted from eq 2 or calculated via
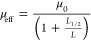
4where *L*_1/2_ is a characteristic channel length at which the contact resistance equals the channel resistance.^19,20^ For our purposes, *L*_1/2_ serves as merely a fitting parameter. An extrapolation of *R*_total_*W* to *L* = 0 yields the channel-width-normalized contact resistance *R*_C_*W* for each value of the gate-overdrive voltage *V*_GS_ – *V*_th_ (in the example in Figure 1: *R*_C_*W* = 69 Ωcm for *V*_GS_ – *V*_th_ = −2 V). The gate-overdrive voltage corresponds to the density of mobile charge carriers in the channel via *n* = |*C*_diel_(*V*_GS_ – *V*_th_)|/*q*, where *q* is the elementary charge. According to eq 1, the slope of the *R*_total_*W* versus *L* relation yields the sheet resistance *R*_sheet_.

Note that the fitting procedure performed using eq 2 is inherently associated with a statistical error (or uncertainty) σ, which is connected to the quality of the fit *R*^2^. This uncertainty is illustrated in Figure 1d as a confidence interval that represents possible deviations from the extracted value *R*_C_*W* ± σ. In the example of Figure 1, it amounts to (69 ± 5) Ωcm.

### Reliability of the TLM Analysis

**Figure 2:**
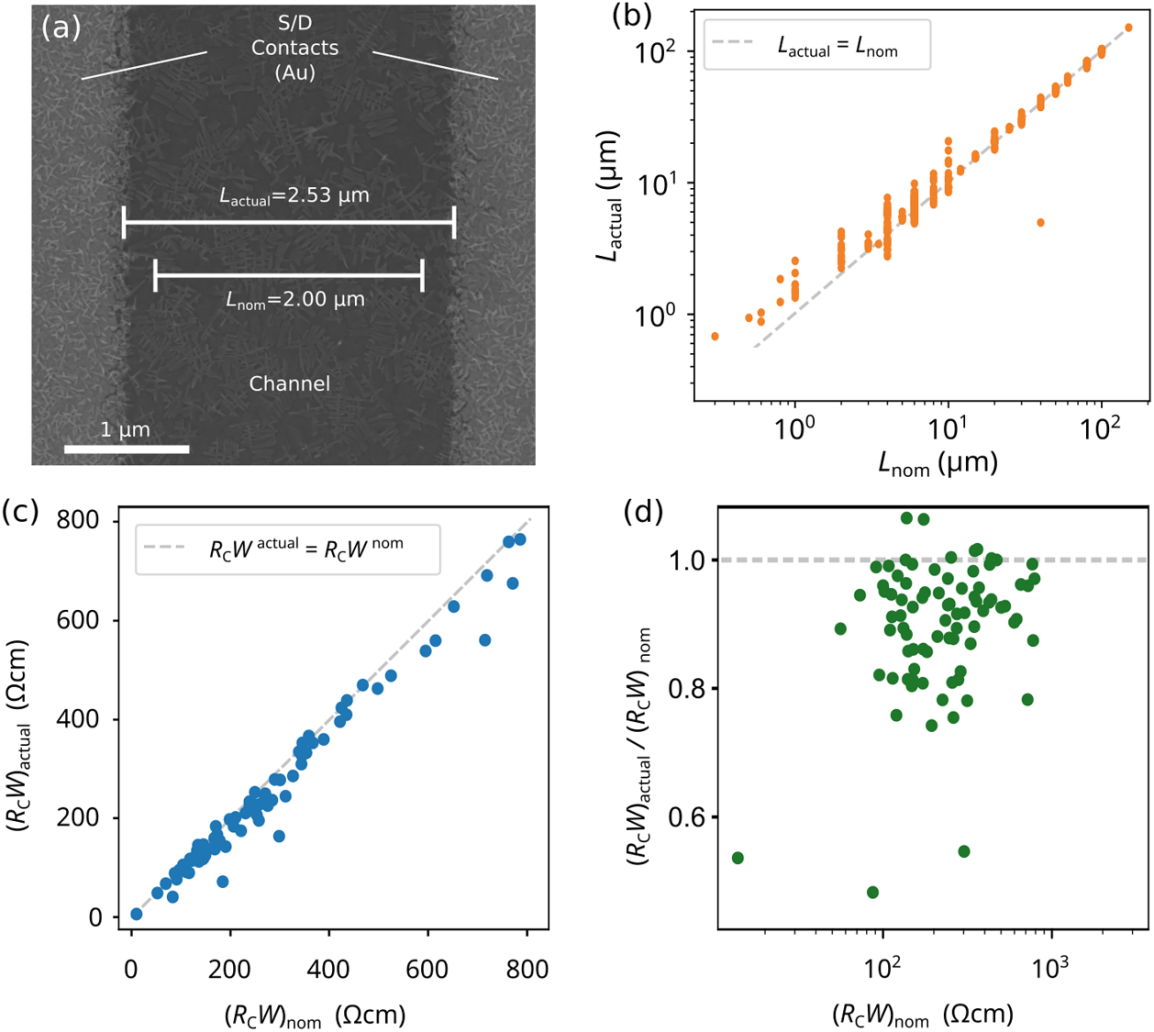
(a) SEM image of a TFT with Au source/drain contacts patterned by stencil lithography. The nominal channel length (*L*_nom_) is 2 μm, but the SEM image indicates that the actual channel length (*L*_actual_) is 2.53 μm, i.e., Δ*L* = *L*_actual_ – *L*_nom_ = 0.53 μm. (b) Actual channel length *L*_actual_ of over 1100 TFTs (measured by SEM) plotted versus the nominal channel length *L*_nom_. For most TFTs, the actual channel length is larger than the nominal channel length by about 0.6 μm. (c) The value that is extracted for the contact resistance of DPh-DNTT TFTs when the actual channel lengths are used in the TLM analysis, (*R*_C_*W*)_actual_, is smaller by about 10% than the value of the contact resistance that is extracted when the nominal channel lengths are used, (*R*_C_*W*)_nom_. (d) The same data plotted in a different manner to better illustrate the relative deviation between (*R*_C_*W*)_actual_ and (*R*_C_*W*)_nom_. The data shown in this figure illustrate that for DPh-DNTTs TFTs fabricated by stencil lithography, *R*_C_*W* will be overestimated by about 10% in case Δ*L* is not properly accounted for in the TLM analysis, regardless of the general magnitude of *R*_C_*W*.

To improve the reliability of the TLM analysis, we included TFTs with a wide range of channel lengths, typically from *L* = 2 to 80 μm. Rather than taking the nominal channel length *L*_nom_ at face value, we determined the actual channel length *L*_actual_ of all TFTs from scanning electron microscopy (SEM) images. The deviation Δ*L* = *L*_actual_ – *L*_nom_ depends on the lithography method employed for the patterning of the source and drain contacts. For the TFTs in this study, the Au source/drain contacts were patterned using stencil lithography.^21^ With this method, the deviation between *L*_actual_ and *L*_nom_ originates mainly from shadowing effects during the vacuum deposition of the source/drain metal through the openings in the mask (see Figure S2).^22,23^ For the TFTs presented here, we found that the median deviation Δ*L* obtained from over 1100 TFTs on over 200 substrates fabricated over the course of three years is +0.6 μm, without any discernible systematic dependence of Δ*L* on the nominal channel length (see Figures 2 and S3). In other words, for the TFT-fabrication process employed here, the actual channel length *L*_actual_ is larger than the nominal channel length *L*_nom_ by about 0.6 μm. If the TLM analysis were performed using the values for the nominal channel length *L*_nom_, rather than the actual channel lengths *L*_actual_, the following systematic error would be introduced to the extracted channel-width-normalized contact resistance:
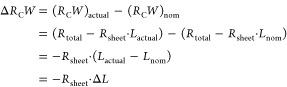
5
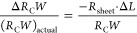
6

In other words, for Δ*L* > 0, *R*_C_*W* would be overestimated (Δ*R*_C_*W* < 0), and for Δ*L* < 0, *R*_C_*W* would be underestimated (Δ*R*_C_*W* > 0), in case the TLM analysis was performed using the nominal channel lengths. For the TFTs presented here, Δ*L* is positive (median +0.6 μm), and *R*_C_*W* would therefore be overestimated (Δ*R*_C_*W* < 0). Note that this systematic error Δ*R*_C_*W* is a different entity than the statistical uncertainty σ associated with the fitting procedure (illustrated in Figure 1d).

Under the assumption that the value of Δ*L* is the same for each TFT, the systematic error Δ*R*_C_*W* can be estimated by using eq 5. However, since Δ*L* is slightly different for each TFT (see Figure S3), a simple addition of eqs 2 and 5 is not sufficient, and the TLM analysis should take into account the individual values of Δ*L* for each TFT. Since the difference between the actual channel length *L*_actual_ and the nominal channel length *L*_nom_ is not systematically dependent on *L*_nom_, the ratio *L*_actual_/*L*_nom_ increases with decreasing *L*_nom_ (see Figure S4); however, this has no implications on the reliability of the TLM analysis.

**Figure 3:**
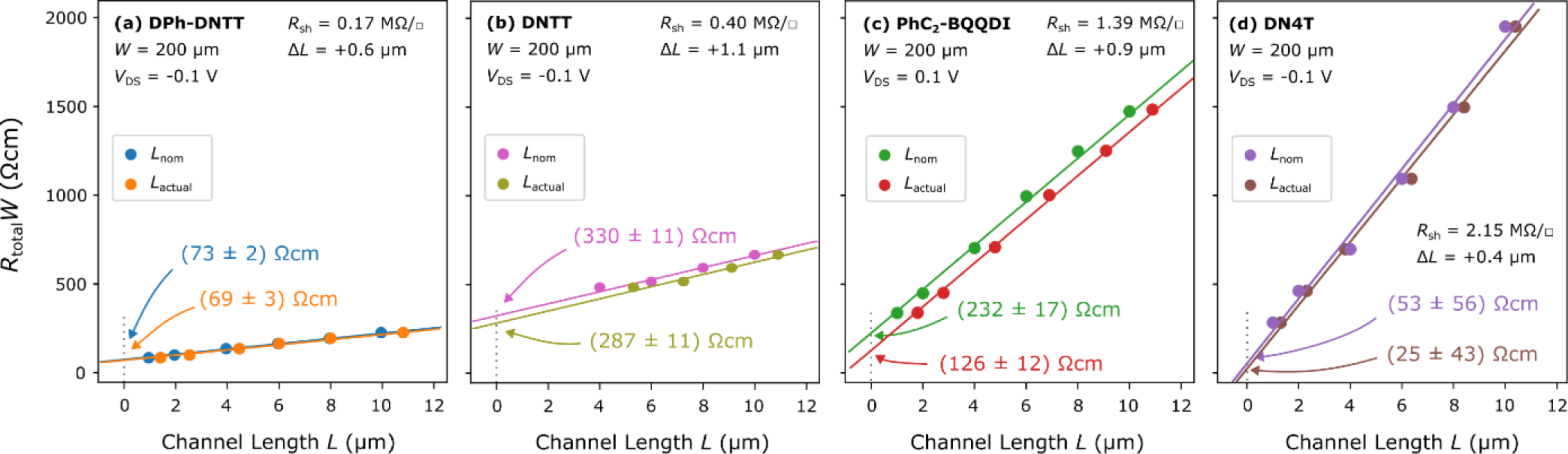
Impact of the sheet resistance of the semiconductor on the reliability of the contact resistance extracted by TLM. Shown are results from TFTs based on (a) DPh-DNTT, (b) DNTT, (c) PhC_2_–BQQDI, and (d) DN4T. For each TLM analysis, the statistical uncertainty σ associated with the fitting procedure, as well as the systematic error Δ*R*_C_*W* that is introduced if the TLM analysis is performed using the nominal instead of the actual channel lengths, is indicated. The magnitude of Δ*R*_C_*W* depends on both Δ*L* and on *R*_sheet_. When *R*_sheet_ is small, Δ*R*_C_*W* is small (e.g., 5% *R*_C_*W* for DPh-DNTT); when *R*_sheet_ is large, Δ*R*_C_*W* is large (and may even exceed the value of *R*_C_*W*, as in the case of DN4T).

Eq 5 indicates that the systematic error Δ*R*_C_*W* depends not only on Δ*L*, but also on the sheet resistance of the semiconductor: the greater *R*_sheet_ is, the more the reliability of the TLM analysis will be compromised if Δ*L* is not taken into account in the TLM analysis. This is illustrated in Figure 3, which shows TLM results from TFTs fabricated by using four different organic semiconductors: DPh-DNTT, DNTT,^24^*N*,*N*′-diphenethyl-3,4,9,10-benzo[*de*]isoquinolino-[1,8-*gh*]quinoline-tetracarboxylic diimide (PhC_2_–BQQDI)^25^ and naphtho[2,3-*b*]thieno-[2‴,3‴:4″,5″]thieno-[2″,3″’:4′,5′]thieno-[3′,2′-*b*]naphtho[2,3-*b*]thiophene (DN4T).^26^ The respective sheet resistances are listed in Table 1; as can be seen, the relative impact of Δ*R*_C_*W* is indeed larger for greater *R*_sheet_. The DPh-DNTT, DNTT, and DN4T TFTs are p-channel transistors, while the PhC_2_–BQQDI TFTs are n-channel transistors. In each graph, we show the TLM analysis performed using the nominal channel lengths, yielding (*R*_C_*W*)_nom_, and the TLM analysis performed using the actual channel lengths, yielding (*R*_C_*W*)_actual_. Comparing the values extracted for (*R*_C_*W*)_nom_ and (*R*_C_*W*)_actual_ in Figure 3 shows that the systematic error Δ*R*_C_*W* = (*R*_C_*W*)_actual_ – (*R*_C_*W*)_nom_ is quite small when the sheet resistance of the semiconductor is small (e.g., DPh-DNTT), but much larger when the sheet resistance is large (in particular for PhC_2_–BQQDI). The systematic error amounts to Δ*R*_C_*W* = −4 Ωcm or about −5% of (*R*_C_*W*)_nom_ for DPh-DNTT; Δ*R*_C_*W* = −43 Ωcm or about −13% for DNTT; Δ*R*_C_*W* = −106 Ωcm or about −46% for PhC_2_–BQQDI; Δ*R*_C_*W* = −28 Ωcm or about −53% for DN4T. Depending on the sheet resistance, the contact resistance would therefore be overestimated significantly in the case that Δ*L* is not properly accounted for in the TLM analysis.

**Table 1:** Summary of the Device Parameters from Figure 3^a^

Organic semiconductor	(*R*_C_*W*)_actual_ ± σ [Ωcm]	μ_0_ [cm^2^/(V s)]	*R*_sheet_ [MΩ/□]	Δ*L* [μm]	Δ*R*_C_*W* [Ωcm]	(Δ*R*_C_*W*)*/* (*R*_C_*W*)_nom_	-*R*_sheet_ Δ*L* [Ωcm]	Λ [μm]
DPh-DNTT	69 ± 3	7.2	0.17	+0.6	–4	–0.05	–10	4.06
DNTT	287 ± 11	3.9	0.40	+1.1	–43	–0.13	–44	7.18
PhC_2_–BQQDI	126 ± 12	1.0	1.39	+0.9	–106	–0.46	–125	0.91
DN4T	25 ± 43	0.9	2.15	+0.4	–28	–0.53	–86	0.12

a Channel-width-normalized contact resistance extracted by performing the TLM analysis using the actual channel lengths (*R*_C_*W*)_actual_; statistical uncertainty σ of the contact resistance associated with the linear regression; intrinsic channel mobility μ_0_; sheet resistance of the semiconductor *R*_sheet_; median deviation between the actual and nominal channel length Δ*L*; systematic error of the contact resistance that would be introduced if the TLM analysis was performed using the nominal, rather than the actual channel lengths (absolute error Δ*R*_C_*W*; relative error (Δ*R*_C_*W*)/(*R*_C_*W*)_nom_); product of the sheet resistance of the semiconductor *R*_sheet_ and the median deviation between the actual and nominal channel length Δ*L* (according to eq 5), this would be the systematic error Δ*R*_C_*W* if Δ*L* was identical for all TFTs); channel length Λ below which the channel resistance *R*_ch_*W* is smaller than the extracted contact resistance *R*_C_*W* (this is the minimum channel length that should be included in the TLM analysis in order to obtain a trustworthy value for the contact resistance).

The sheet resistance of the semiconductor affects not only the systematic error Δ*R*_C_*W* but also the statistical uncertainty σ that arises from the fitting procedure. To illustrate this, the statistical uncertainty from each TLM analysis is indicated in Figure 3. Figure 3a shows that when the sheet resistance is small (0.17 MΩ/□ for DPh-DNTT), the statistical uncertainty σ is also small (amounting to only 4% of *R*_C_*W* in the case of the DPh-DNTT transistors), as expected from theory.^27^ However, in extreme cases in which the contact resistance is small and the sheet resistance of the semiconductor is large (e.g., in the DN4T TFTs with *R*_sheet_ = 2.15 MΩ/□, shown in Figure 3d), the statistical uncertainty can even be larger than the contact resistance ((*R*_C_*W*)_actual_ = 25 Ωcm; σ = 43 Ωcm), which obviously renders the TLM results questionable. For a reliable extraction of the contact resistance in such cases (i.e., when the contact resistance is small and the sheet resistance of the semiconductor is large), the TLM analysis should include TFTs with (actual) channel lengths that are sufficiently small so that the channel resistance of the shortest TFT (*R*_ch_*W* = *R*_sheet_·*L*) is smaller than the contact resistance. (The fact that TFTs with a very small channel length may be contact-limited is irrelevant here, and the TLM analysis will still be valid.)

In Figure 3d, the DN4T TFT with the smallest channel length (*L*_actual_ = 1.3 μm) has a channel resistance of *R*_ch_*W* = 280 Ωcm, which is considerably larger than the extracted contact resistance of *R*_C_*W* = (25 ± 43) Ωcm, rendering the value of the contact resistance unreliable. To obtain a meaningful value for the contact resistance of these TFTs, we would need to fabricate TFTs with a channel length of *L*_actual_ < Λ = (*R*_*C*_*W*)_actual_/*R*_*sheet*_ = 0.12 μm. Since this is beyond the capabilities of stencil lithography,^21^ we have to accept the smallest measured total resistance (*R*_total_*W* = 280 Ωcm for *L*_actual_ = 1.3 μm) as the upper limit for the contact resistance of these DN4T TFTs, even though this might grossly overestimate the actual contact resistance. For comparison, the DPh-DNTT TFT with the smallest channel length in Figure 3a (*L*_actual_ = 1.4 μm) has a channel resistance *R*_ch_*W* of 88 Ωcm, which is comparable to the extracted contact resistance (*R*_C_*W* = 69 Ωcm ±3 Ωcm), implying that this particular value of the contact resistance can be trusted.

### Substrate-to-Substrate Variation of the Contact Resistance

**Figure 4:**
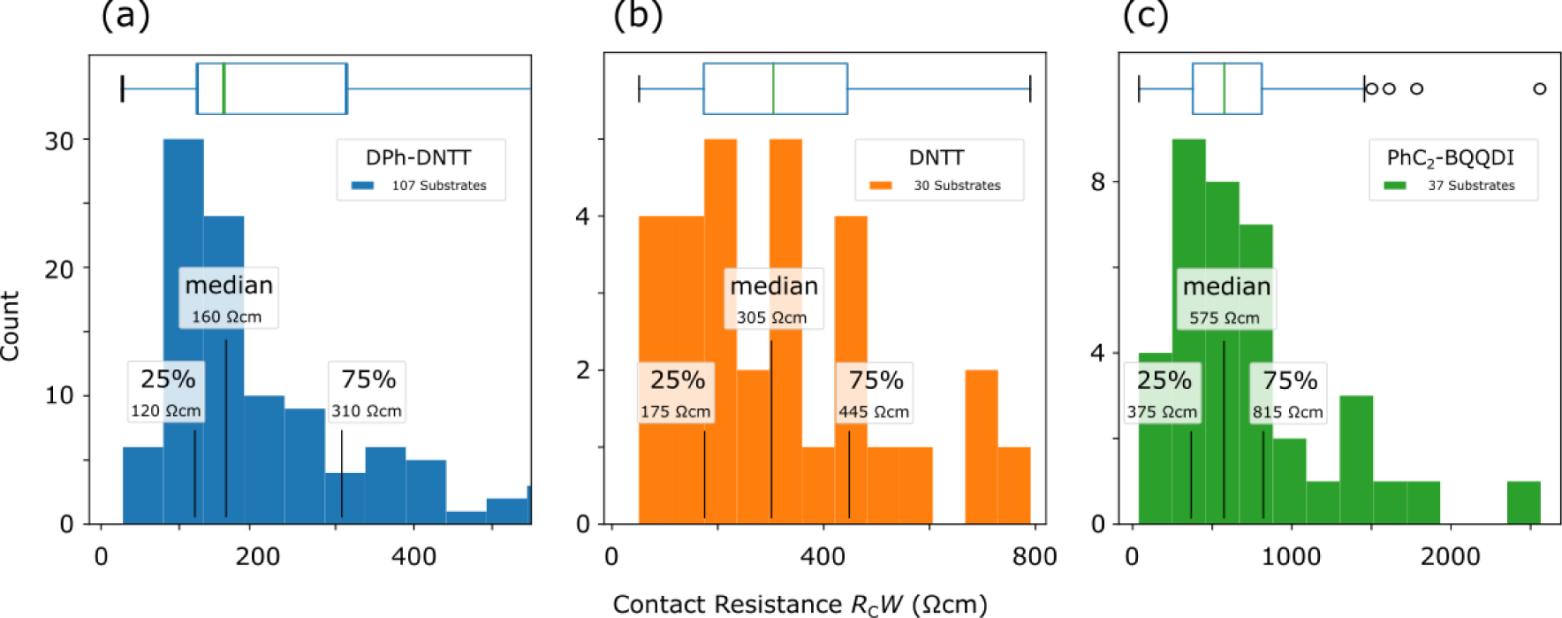
Histograms of the channel-width-normalized contact resistance *R*_C_*W* of a large number of DPh-DNTT p-channel TFTs (a), DNTT p-channel TFTs (b), and PhC_2_–BQQDI n-channel TFTs (c), all fabricated in the bottom-gate, bottom-contact (inverted coplanar) device architecture. The median values and the 25% and 75% percentiles of the distribution are (160 ^+150^_–40_) Ωcm for the DPh-DNTT TFTs, (305 ^+140^_–130_) Ωcm for the DNTT TFTs, and (575 ^+240^_–200_) Ωcm for the PhC_2_–BQQDI TFTs. The substrates were fabricated over a period of three years, and the TLM measurements were conducted within two hours of device fabrication. On each of the 174 substrates, at least five (most often eight) TFTs with channel lengths ranging from at least 4–50 μm were measured.

Over a period of three years, we fabricated several hundred substrates with nominally identical TFTs and extracted the contact resistance by TLM, as described above. In doing so, we found a noticeable spread of the contact resistance, despite the fact that the TFTs were fabricated using the same materials and processes, and despite the fact that the measurements were always performed within two hours after completion of the TFT-fabrication process. The histograms in Figure 4 show a relatively broad, mostly asymmetric distribution of the contact resistance of bottom-contact TFTs based on three different organic semiconductors (DPh-DNTT, DNTT, PhC_2_–BQQDI). The smallest contact resistances we observed within this collection of substrates are (25 ± 6) Ωcm for DPh-DNTT, (55 ± 8) Ωcm for DNTT, and (160 ± 105) Ωcm for PhC_2_–BQQDI. These values are close to (or below) the lowest contact resistances reported for TFTs based on each of these organic semiconductors.^17,28,29^ The median values of the contact resistance we have measured on these 174 substrates are 160 Ωcm for DPh-DNTT, 305 Ωcm for DNTT, and 575 Ωcm for PhC_2_–BQQDI. In other words, the median values of the contact resistance are considerably larger than the smallest values measured for each of these three semiconductors, and we find a relatively large spread from the median values for each semiconductor. According to the 25% and 75% percentiles of the distribution, this spread amounts to ^+150^_–40_ Ωcm for DPh-DNTT, ^+140^_–130_ Ωcm for DNTT, and ^+240^_–200_ Ωcm for PhC_2_–BQQDI. Note that this spread is not limited to TFTs fabricated in the bottom-contact device architecture: Top-contact TFTs fabricated for comparison exhibit a similarly large variability (Figure S5). Oftentimes in literature, only the best results obtained in each study are reported, making it difficult to benchmark the substrate-to-substrate variability that we are reporting here to the variability seen by other groups.

### Statistical Analysis of the Contact Resistance and Correlation with Environmental Factors

It is well-known that the performance parameters of transistors based on organic semiconductors are heavily influenced by environmental factors. To investigate the origin of the substrate-to-substrate variations that we have observed in the contact resistance of our nominally identical organic TFTs (see Figure 4), we therefore performed a statistical analysis of the results obtained from over 100 substrates with respect to some of the environmental conditions at the time of fabrication of these TFTs. Geiger et al. recently showed a correlation between the substrate temperature during the deposition of the gate metal and the resulting surface roughness of the gate metal, which affects the thin-film morphology of the organic semiconductor and thus the TFT characteristics.^30^ Kang et al. reported on the influence of the substrate temperature during the deposition of DPh-DNTT on the carrier mobility of the TFTs.^31^ Lamport et al. studied the correlation between the metal-deposition rate and the contact resistance.^15^ Other studies evaluated the impact of oxygen and humidity on the performance and stability of organic TFTs.^32−34^ In all of these studies, the process parameters were varied intentionally, but it is certainly conceivable that the TFT characteristics are also affected by unintentional parameter variations. For the present study, we kept all fabrication-process parameters that we are able to control reliably and with good accuracy (choice and purity of materials, substrate temperature and deposition rate during vacuum depositions, film thicknesses, solution concentration and immersion times for surface functionalization, air temperature in the lab, intensity and color of illumination in the lab, etc.) constant, while monitoring a number of environmental parameters that are subject to unintentional variations. The values that we chose for the readily controllable fabrication-process parameters (substrate temperature, deposition rate, film thickness, solution concentration, immersion time) are the values that we had previously determined to be the optimum. For example, while we are able to control the air temperature in the laboratory with negligible deviation (20 °C ± 1 °C), other conditions are subject to more significant unintentional fluctuations, in particular the relative humidity (rH) in the lab and the base pressure in the vacuum system for the deposition of the organic semiconductor (*p*_OSC_) and the Au source/drain contacts (*p*_contact_). The humidity varies between about 30% and 65% over the course of a year, and the base pressure varies between about 10^–7^ and 10^–5^ mbar, depending on, for example, the quality of the vacuum seal.

**Figure 5:**
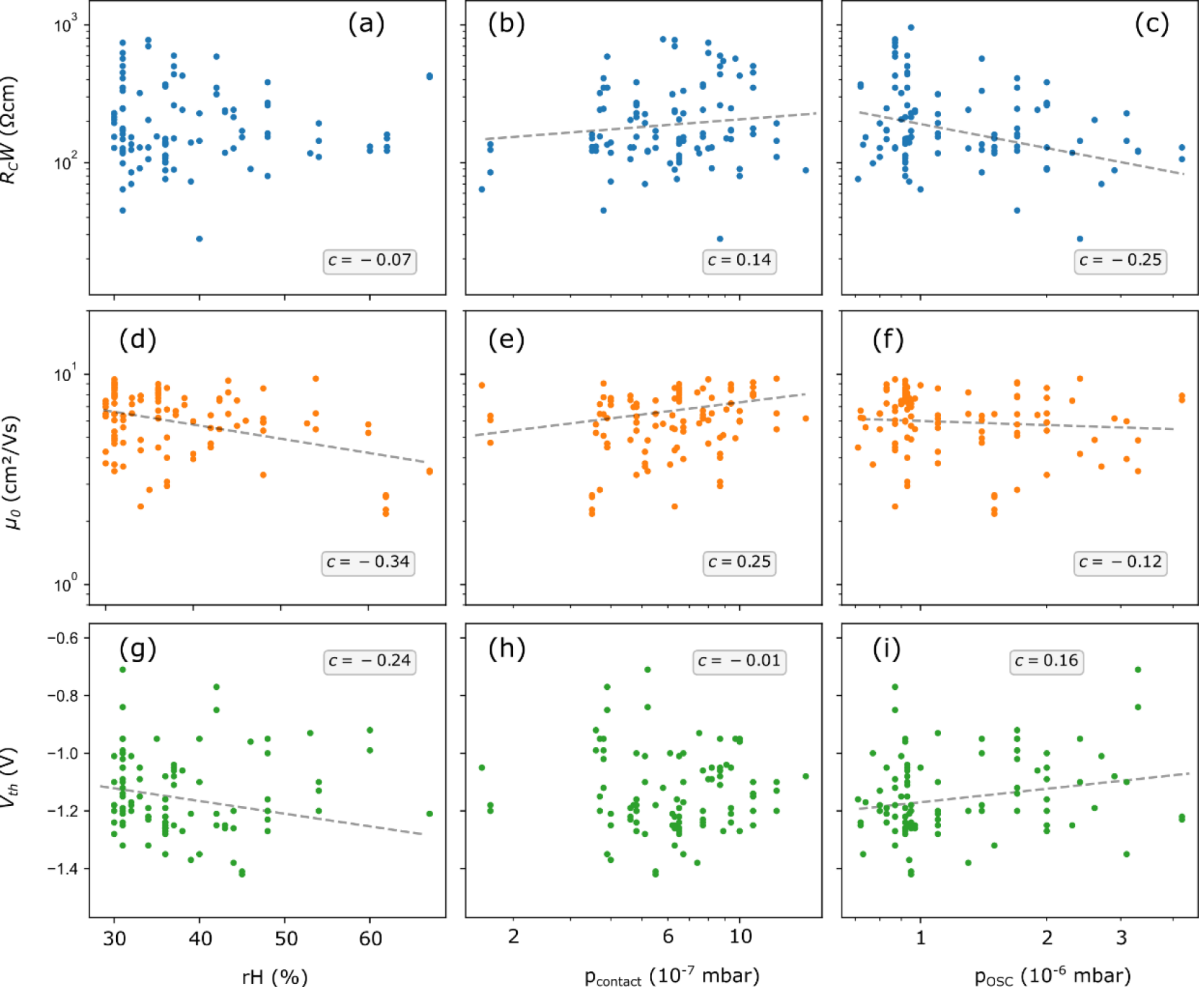
Statistics of the channel-width-normalized contact resistance *R*_C_*W* (a–c), the intrinsic channel mobility μ_0_ (d–f), and the threshold voltage *V*_th_ (g–i) of more than 1000 DPh-DNTT TFTs fabricated in the bottom-gate, bottom-contact (inverted coplanar) device architecture on more than 100 substrates over a period of three years. The parameters were extracted from electrical measurements performed within two hours after device fabrication. They are plotted versus the relative humidity in the laboratory during device fabrication rH (a,d,g), versus the base pressure in the vacuum system during the deposition of the organic semiconductor *p*_OSC_ (b,e,h), and versus the base pressure in the vacuum system during the deposition of the source/drain contacts *p*_contact_ (c,f,i). Each data point for *V*_th_ represents an average of all TFTs measured on a single substrate. Dashed lines represent fits to the data. The *R*^2^ values of these fits are very small, between 0.02 and 0.1, which reinforces the weakness of the correlations.

Figure 5 shows how the contact resistance *R*_C_*W*, the intrinsic channel mobility μ_0_, and the threshold voltage *V*_th_ of DPh-DNTT TFTs fabricated in the bottom-gate, bottom-contact (inverted coplanar) device architecture over a period of three years vary with unintentional variations of rH, *p*_OSC_, and *p*_contact_. Note that of these three performance parameters, the contact resistance *R*_C_*W* exhibits a much larger variation than μ_0_ and *V*_th_, despite the fact that for each TLM analysis we included TFTs with a very small nominal channel length (4 μm in most cases, 2 μm in some cases). The correlation coefficients *c* in the Pearson correlation matrix that was calculated from the measurement data are shown in each graph and are listed in Table 2. We consider here correlations with |*c*| < 0.1 to be negligible, 0.1 < |*c*| < 0.25 to be weak, 0.25 < |*c*| < 0.4 to be moderate, and |*c*| > 0.4 to be strong. Table 2 indicates that there are no strong correlations between any of the parameters. For several pairs of performance parameters and environmental conditions, a weak or moderate correlation can be seen. For example, the contact resistance correlates positively with *p*_contact_ (Figure 5b; *c* = 0.14) and negatively with *p*_OSC_ (Figure 5c; *c* = −0.25); the intrinsic channel mobility correlates negatively with the relative humidity in the lab (Figure 5d; *c* = −0.34), as well as with *p*_OSC_ (Figure 5f; *c* = −0.12), and positively with *p*_contact_ (Figure 5e; *c* = −0.25); the threshold voltage correlates negatively with rH (Figure 5g; *c* = −0.24) and positively with *p*_OSC_ (Figure 5i; *c* = 0.16).

**Table 2:** Correlation Matrix for the Process Parameters^a^ and the TFT Parameters^b^ Obtained from the Fabrication of DPh-DNTT TFTs

	rH	*p*_contact_	*p*_OSC_	*R*_C_*W*	μ_0_	*V*_th_
**rH**	1	–0.06	0.04	–0.07	–0.34	–0.24
***p*_contact_**		1	–0.12	0.14	0.25	–0.01
***p*_OSC_**			1	–0.25	–0.12	0.16
***R*_C_*W***				1	–0.21	–0.14
**μ**_**0**_					1	–0.04
***V*_th_**						1

a Relative humidity in the laboratory rH, base pressure in the vacuum systems during the deposition of the contacts *p*_contact_, and the organic semiconductor *p*_OSC_.

b Channel-width-normalized contact resistance *R*_C_*W*, intrinsic channel mobility μ0, and threshold voltage *V*_th_.

To some extent, these correlations can be rationalized. For example, a cleaner environment during the deposition of the functional materials can lead to a smaller density of defects—both at the contact-semiconductor interface and within the semiconducting layer—and thus to a smaller *R*_C_*W* and to a greater μ_0_.^35^ This is consistent with the trends seen in Figure 5b,f, although the trends observed here are quite weak. With the same rationale, a trend toward smaller *R*_C_*W* is expected for lower *p*_OSC_, but such a trend is neither apparent from the measurement data in Figure 5c nor from the respective correlation coefficient.

The trends observed for the threshold voltage (Figure 5g–i) are contradictory. A smaller defect density (i.e., a lower humidity and a lower base pressure) may be expected to bring the threshold voltage closer to zero. This is indeed seen in Figure 5g (*V*_th_ is shifted closer to 0 V for smaller rH), but it is observed in neither Figure 5h (no significant trend observed) nor Figure 5i (*V*_th_ is shifted away from 0 V for smaller *p*_OSC_). The reasons for this behavior are unclear. Since we found no correlation with a single fabrication parameter (Table 1) to be sufficiently strong to explain the very large spread in the contact resistance, we believe that these deviations are largely stochastic. The observation that the contact resistance of our DPh-DNTT TFTs does not show a strong dependence on the base pressure during the metal and organic-semiconductor depositions can perhaps be taken as confirmation that a base pressure below about 1 × 10^–6^ mbar is sufficient for these process steps. The observation that the impact of the humidity on the contact resistance is so weak is more surprising, and we are unable to offer a convincing explanation at this point.

In addition to correlations between environmental and device parameters, we can use the measurement data to identify correlations between any two device parameters (see Table 1). For example, a larger intrinsic channel mobility leads to a smaller contact resistance (*c* = −0.21). This is expected, since a larger intrinsic channel mobility results in a larger space-charge limited current in the regions near the contact-semiconductor interface and thereby to a smaller contact resistance (see also Figure S6).^36^ Furthermore, a larger μ_0_ indicates a better thin-film morphology of the organic semiconductor, which in turn has been shown to be highly beneficial for a small contact resistance in inverted coplanar TFTs.^3,13^ The correlation between *V*_th_ and *R*_C_*W* is quite weak (*c* = 0.14), which supports the hypothesis that the threshold voltage is a parameter that is dictated mainly by the properties of the gate dielectric and the semiconductor-dielectric interface, whereas the contact resistance is dictated mainly by the properties of the contacts and the contact-semiconductor interface.

The TFTs shown in Figure 5 were fabricated on a large number of substrates over a period of three years. Each of these substrates represents an individual fabrication run, and although we kept all controllable fabrication parameters constant, unintended process-parameter variations are inevitable. To complement our findings about the substrate-to-substrate variations of important device parameters corresponding to this large set of fabricated TFTs, we also evaluate how the TFT characteristics vary between substrates that have been fabricated simultaneously within the same process run (see Figure S7). These substrates were loaded into the deposition system together and mounted onto the substrate holder side-by-side for each deposition (gate electrodes, source/drain contacts, organic semiconductor). We find that on six substrates with DPh-DNTT p-channel TFTs, the contact resistances are all very similar to one another, falling into the range of (113 ± 14) Ωcm. This amounts to a variability of about 12% within the same batch. A similar value is observed for nine simultaneously fabricated substrates with PhC_2_–BQQDI n-channel TFTs, where a contact resistance of (595 ± 91) Ωcm indicates a variability of 16% within the same batch.

Although this variability within a single batch is not negligible, it is significantly smaller than the value of more than 100% that was observed for the batch-to-batch variability shown in Figure 4. For a reliable expectation value of the contact resistance, it is therefore preferable to consider data from multiple fabrication runs to ensure a valid comparison between modifications in TFT fabrication that may reduce the contact resistance.

## Conclusion

The contact resistance is one of the most important performance parameters of organic TFTs, and even relatively small enhancements can be challenging to achieve.^3^ The reliable extraction of *R*_*C*_*W* using TLM analysis is therefore critically important. This reliability can be greatly enhanced in two different ways: first, by measuring the actual channel lengths of the transistors, rather than relying on the nominal channel-length values; and second, by including transistors with very small channel lengths in the TLM analysis, so that the channel resistance of the transistor with the smallest channel length is smaller than the contact resistance. How small this minimum channel length needs to be depends on the sheet resistance of the semiconductor: the larger the sheet resistance, the smaller the minimum channel length Λ = (*R*_*C*_*W*)_actual_/*R*_*sheet*_ needs to be for reliable extraction of the contact resistance.

The contact resistance of organic TFTs varies greatly from one fabrication run to the next (and even on substrates fabricated within the same fabrication run), no matter how much care is taken to keep all materials and process parameters the same. This is true regardless of the choice of the organic semiconductor, regardless of the device architecture (coplanar or staggered), and regardless of the magnitude of the contact resistance. The substrate-to-substrate variation in the contact resistance is notably larger than the variation in other TFT parameters (charge-carrier mobility, threshold voltage, etc.). There is no strong correlation between the contact resistance and the environmental parameters present during TFT fabrication, such as the humidity in the laboratory or the base pressure in the vacuum system during the deposition of the source/drain contacts and the organic-semiconductor layer. This leads us to believe that the large spread that was found over a period of several years is mainly of a stochastic nature.

## Experimental Section

### Materials

- 2,9-diphenyl-dinaphtho[2,3-*b*:2′,3′-*f*]thieno[3,2-*b*]thiophene (DPh-DNTT) was kindly provided by K. Ikeda, Y. Sadamitsu, and S. Inoue (Nippon Kayaku, Japan).
- Dinaphtho[2,3-*b*:2′,3′-*f*]thieno[3,2-*b*]thiophene (DNTT) was purchased from Sigma-Aldrich (Germany).
- Diphenylethyl-3,4,9,10-benzo[*de*]isoquinolino[1,8-*gh*]quinolinetetracarboxylic diimide (PhC_2_–BQQDI) was purchased from Fujifilm Wako Pure Chemical Cooperation (Neuss, Germany).
- Naphtho[2,3-*b*]thieno-[2‴,3‴:4″,5″]thieno-[2″,3″:4′,5′]thieno-[3′,2′-*b*]naphtho[2,3-*b*]thiophene (DN4T) was kindly provided by Yves Geerts, Université Libre de Bruxelles.
- *N*,*N*′-bis(2,2,3,3,4,4,4-fluorobutyl)-(1,7 and 1,6)-dicyano-perylene-tetracarboxylic diimide (ActivInk N1100) was procured from Polyera Corp. (Skokie, IL, U.S.A.).
- *n*-Tetradecylphosphonic acid was purchased from PCI Synthesis (Newburyport, MA, U.S.A.).
- Pentafluorobenzenethiol (PFBT) and 4-(methylsulfanyl)-thiophenol (MeSTP) were purchased from TCI Deutschland GmbH (Eschborn, Germany).

### TFT Fabrication

All TFTs were fabricated on doped-silicon substrates. In the first step, a 30 nm-thick layer of aluminum is deposited by thermal evaporation in a vacuum at a base pressure of ∼10^–7^ mbar and with a rate of 2.5 nm/s.^30^ The aluminum layer is not patterned and serves as a common gate electrode for all TFTs on the substrate. The gate dielectric is a stack of aluminum oxide (obtained by exposing the aluminum to oxygen plasma) and a self-assembled monolayer of *n*-tetradecylphosphonic acid (obtained by immersing the substrate in a 2-propanol solution of the phosphonic acid). It has a total thickness of about 8 nm and a unit-area capacitance of 0.6 μF/cm^2^.^37^ To define the source and drain contacts, gold with a thickness of 25–30 nm is deposited by thermal evaporation in a vacuum and with a deposition rate of 0.03 nm/s^15^ through a silicon stencil mask.^38^ The surface of the Au source and drain contacts is then functionalized with a chemisorbed monolayer of pentafluorobenzenethiol (PFBT; for the p-channel TFTs)^13^ or methylthiothiophenol (MeSTP; for the n-channel TFTs)^39^ by immersing the substrate into a 10 mMol ethanol solution of the thiol. In the final process step, a nominally 30 nm-thick layer of the organic semiconductor is deposited by thermal sublimation in a vacuum with a deposition rate of 0.04 nm/s. During semiconductor deposition, the substrate is held at a constant temperature of nominally 90 °C (for the p-channel TFTs) or 140 °C (for the n-channel TFTs).

For comparison, we also fabricated TFTs in the bottom-gate, top-contact (inverted staggered) device architecture, in which case the deposition of the organic semiconductor was carried out prior to the deposition of the source/drain contacts, and no thiol treatment was performed. The air temperature in the laboratory in which all fabrication-process steps were conducted is actively controlled to a value of (20 ± 1) °C.

### Electrical Characterization

On each substrate, at least 50 TFTs with at least 10 different channel lengths, typically ranging from 2 to 100 μm, are available for electrical characterization. For each TLM analysis, usually 7–9 different channel lengths are taken into account, with the minimum being 5 different channel lengths. For all substrates shown here, the shortest channel length is less than 5 μm. The current–voltage characteristics of the TFTs were recorded using an Agilent 4156C Semiconductor Parameter Analyzer controlled remotely using the software “SweepMe!” (https://sweep-me.net) at a temperature of 20 ± 1 °C in ambient air (with a humidity ranging from 28% to 65%, depending on the time of year) under weak yellow laboratory light.

## Supporting Information Available

Relevant energy levels of the materials used, schematic explaining the shadowing process, statistics of the real channel length depending on the nominal values, histogram of the contact resistance in top-contact n-channel TFTs, correlation between intrinsic mobility and contact resistance, variation of the contact resistance within the same fabrication run (PDF)
