# Supplementary material for: Reliability of the Transmission Line Method and Reproducibility of the Measured Contact Resistance of Organic Thin-Film Transistors
Source: ACS Nano. 2025 Mar 10;19(10):9915–24. doi: 10.1021/acsnano.4c15828 (PMC11924322; doi:10.1021/acsnano.4c15828)
Supplement: Supplementary file 1 — nn4c15828_si_001.pdf [file nn4c15828_si_001.pdf]

Supporting Information

Reliability of the Transmission Line Method and Reproducibility of the Measured Contact Resistance of Organic Thin-Film Transistors

Tobias Wollandt[†]*, Sabrina Steffens*[†]*, Yurii Radiev*[‡]*, Florian Letzkus*[₴]*, Joachim N. Burghartz*[₴]*, Gregor Witte*[‡]*, Hagen Klauk*[†]* *

[†] Max Planck Institute for Solid State Research, Heisenbergstr. 1, 70569 Stuttgart, Germany

[‡] Molecular Solids Group, Philipps-Universität Marburg, Renthof 7, 35032 Marburg, Germany

[₴] Institute for Microelectronics Stuttgart (IMS CHIPS), Allmandring 30A, 70569 Stuttgart, Germany

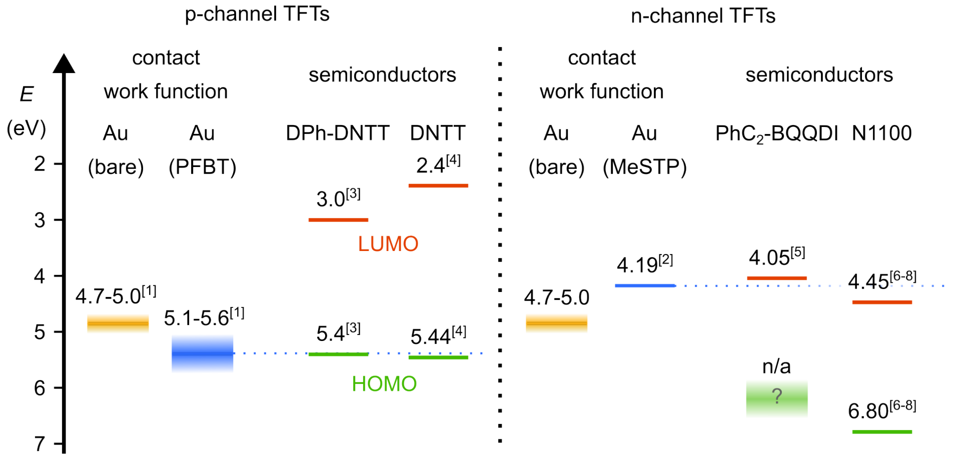

Figure S1. Work function of the Au source/drain contacts with and without thiol functionalization; highest occupied molecular orbitals (HOMO) of the semiconductors in the p-channel TFTs; lowest unoccupied molecular orbitals (LUMO) of the semiconductors in the n-channel TFTs. The values were taken from the following publications:

(1) Wollandt, T.; Letzkus, F.; Burghartz, J.N.; Klauk, H.: Comparative Study of Silver and Gold Source/Drain Contacts for Organic Thin-Film Transistors with Low Contact Resistance. *Adv. Electron. Mater.* **2024**, *10*, 2300841

(2) Boudinet, D.; Benwadih, M.; Yabin, Q.; Altazin, S.; Verilhac, J.-M.; Kroger, M.; Serbutoviez, C.; Gwoziecki, R.; Coppard, R.; Le Blevennec, G.; Kahn, A.; Horowitz, G.: Modification of Gold Source and Drain Electrodes by self-assembled Monolayer in Staggered n- and p-Channel Organic Thin Film Transistors. *Org. Electronics* **2010**, *11*, 227-237

(3) Zhou, Z.; Wu, Q.; Cheng, R.; Zhang, H.; Wang, S.; Chen, M.; Xie, M.; Chan, P.K.L.; Grätzel, M.; Fenghou, S.-P.: Orientation-Engineered Small-Molecule Semiconductors as Dopant-Free Hole Transporting Materials for Efficient and Stable Perovskite Solar Cells. *Adv. Funct. Mater.* **2021**, *31*, 2011270

(4) Yamamoto, T. and Takimiya, K.: Facile Synthesis of Highly π-Extended Heteroarenes, Dinaphtho[2,3-b:2‘,3‘-f]chalcogenopheno[3,2-b]chalcogenophenes, and Their Application to Field-Effect Transistors, *J. Am. Chem. Soc.* **2007**, *129*, 2224-2225

(5) Yu, C.P.; Kojima, N.; Kumagai, S.; Kurosawa, T.; Ishii, H.; Watanabe, G.; Takeya, J.; Okamoto, T.: Approaching isotropic charge transport of n-type organic semiconductors with bulky substituents. *Commun. Chem.* **2021,** *4*, 155

(6) Jones, B.A.; Ahrens, M.J.; Yoon, M.-H.; Facchetti, A.; Marks, T.J.; Wasielewski, M.R.: High-Mobility Air-Stable n-Type Semiconductors with Processing Versatility: Dicyanoperylene-3,4:9,10-bis(dicarboximides). *Angew. Chem. Int. Ed.* **2004**, *43*, 6363-6366

(7) Barra, M.; Chiarella, F.; Chainese, F.; Vaglio, R.; Cassinese, A.: Perylene-Diimide Molecules with Cyano Functionalization for Electron-Transporting Transistors. *Electronics* **2019**, *8*, 246

(8) Jones, B.A.; Facchetti, A.; Wasielewski, M.R.; Marks, T.J.: Tuning Orbital Energetics in Arylene Diimide Semiconductors. Materials Design for Ambient Stability of n-Type Charge Transport. *J. Am. Chem. Soc.* **2007**, *129*, 1525

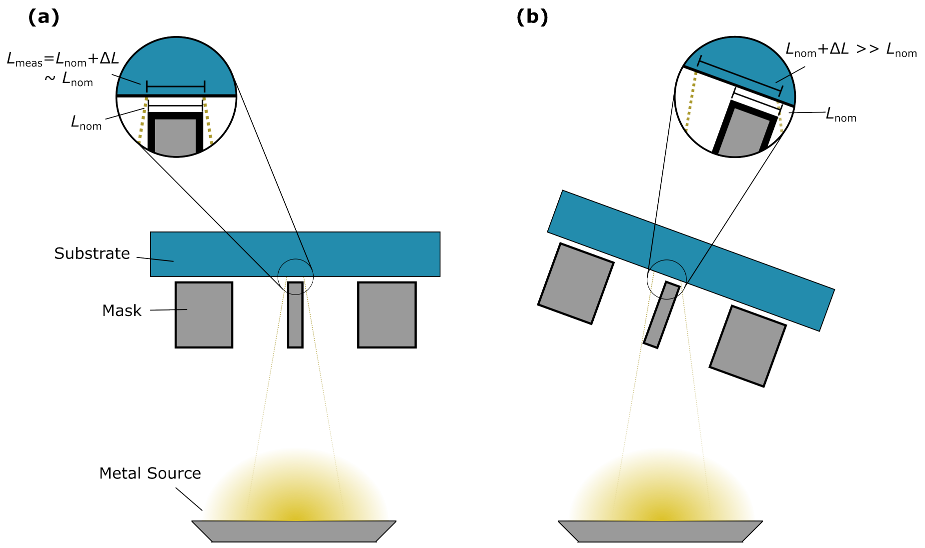

Figure S2. Schematic of the geometry during the vacuum deposition of the source/drain contacts through the openings in the stencil mask. (a) If the substrate plane is parallel to the metal-evaporation source, the deviation ΔL between the actual and the nominal channel length is determined only by the (unavoidable) gap between the stencil mask and the substrate. In this case, ΔL will usually be negative ($\Delta L < 0$; see reference 22 of the main text). (b) If the substrate is tilted (even if the tilt angle is extremely small; note that in the schematic drawing, the tilt angle is greatly exaggerated), ΔL can be positive. Since the stencil masks employed here have a relatively large thickness (20 µm) compared to the smallest channel lengths (~1 µm), the relative deviation $\Delta L/L_{nom}$ can be quite large, up to 100% for a tilt angle of 1 to 2° and a channel length of 1 µm (see Figure S3).

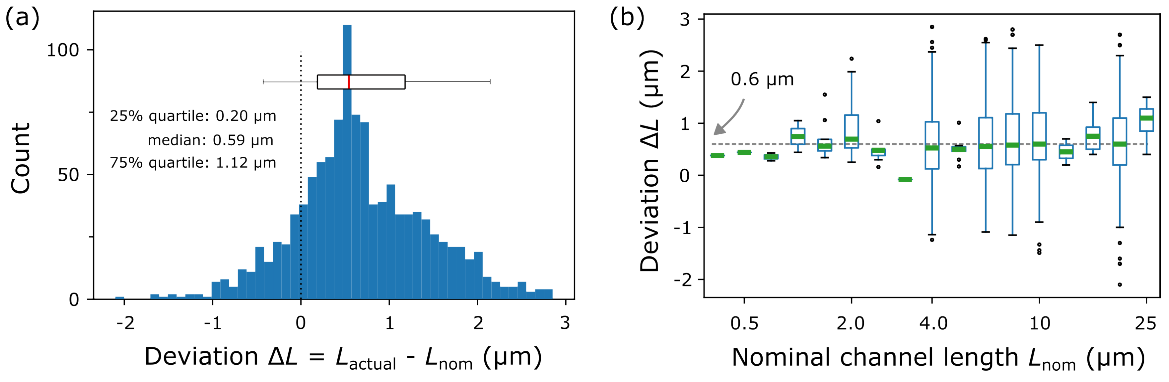

Figure S3. (a) Histogram of the deviation ΔL between the actual and the nominal channel length measured for about 1100 TFTs fabricated on 200 substrates. The median is ΔL = 0.59 µm, and the 25% and 75% quartiles are $^{+0.53}_{-0.39}$ µm. (b) Distribution of ΔL plotted versus the nominal channel length. The boxes extend from the Q1 to Q3 quartile values of the data, with a line at the median (Q2). The whiskers extend from the edges of box to show the range of the data. By default, they extend no more than *1.5 * IQR (IQR = Q3 - Q1)* from the edges of the box, ending at the farthest data point within that interval. Outliers are plotted as separate dots.

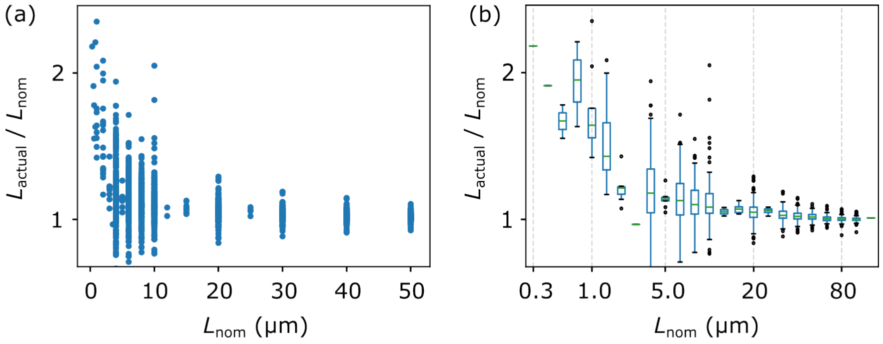

Figure S4. Ratio between the actual channel length L_{actual} and the nominal channel length L_{nom}, determined from SEM images of over 1100 TFTs and plotted versus the nominal channel length L_{nom}. Since the difference between L_{actual} and L_{nom} is not systematically dependent on L_{nom} (see Figure S2), the ratio L_{actual}/ L_{nom} increases with decreasing L_{nom}; however, this has no implications on the reliability of the TLM analysis. The raw data is shown as a scatter plot (a), and its distribution is shown as a box plot (b). The boxes extend from the Q1 to Q3 quartile values of the data, with a line at the median (Q2). The whiskers extend from the edges of box to show the range of the data. By default, they extend no more than *1.5 * IQR (IQR = Q3 - Q1)* from the edges of the box, ending at the farthest data point within that interval. Outliers are plotted as separate dots.

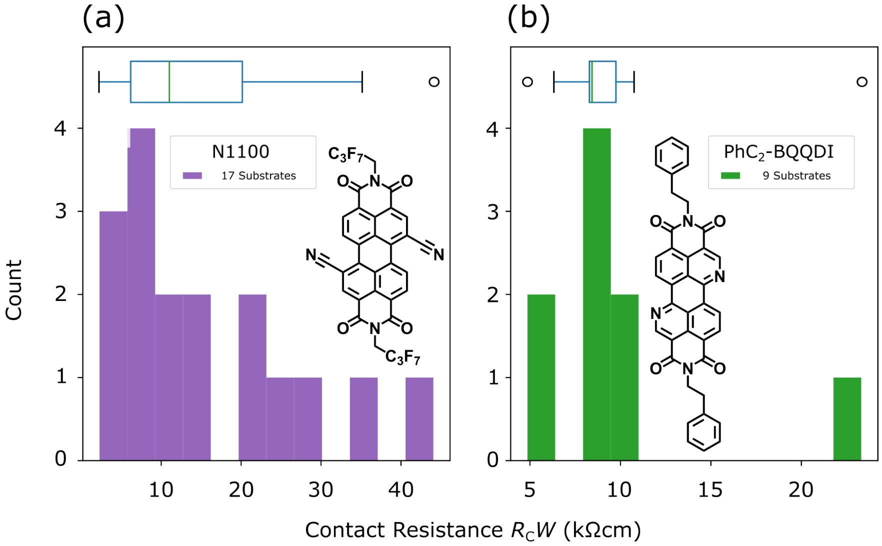

Figure S5. Histograms of the channel-width-normalized contact resistance R_CW of 26 sets of n-channel TFTs fabricated in the bottom-gate, top-contact (inverted staggered) device architecture using the organic semiconductors (a) N,N'-bis(2,2,3,3,4,4,4-fluorobutyl)-(1,7 & 1,6)-dicyano-perylene-tetracarboxylic diimide [N1100, Jones, Angew. Chem. Int. Ed., vol. 43, p. 6363, 2004] and (b) PhC_2-BQQDI.

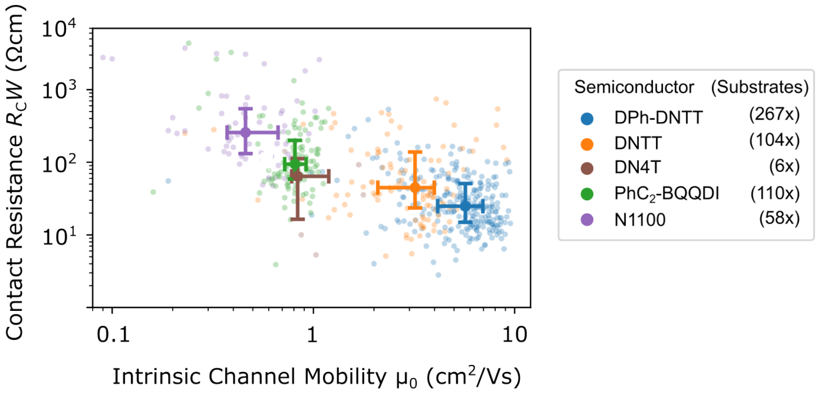

Figure S6. Correlation between the channel-width-normalized contact resistance R_CW and the intrinsic channel mobility μ_0 of TFTs fabricated in the bottom-gate, bottom-contact (inverted coplanar) device architecture. Each color represents a different organic semiconductor. Each light-colored data point represents one set of (at least five) TFTs on an individual substrate, in total over 500 substrates over the course of three years. The dark-colored data points and error bars indicate the median value and (25% to 75%) interquartile range over all substrates for each semiconductor. This graph illustrates that a larger intrinsic channel mobility leads to a smaller contact resistance.

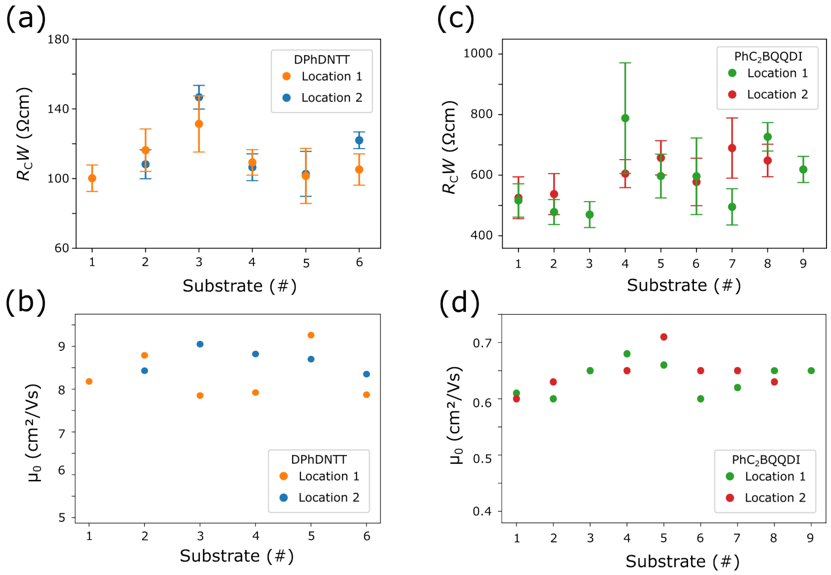

Figure S7. (a,b) Channel-width-normalized contact resistance R_CW and intrinsic channel mobility μ_0 of DPh-DNTT TFTs fabricated simultaneously on six substrates using identical process conditions. (c,d) Contact resistance R_CW and intrinsic channel mobility μ_0 of PhC_2-BQQDI TFTs fabricated simultaneously on nine substrates using identical conditions. All TFTs were fabricated in the bottom-gate, bottom-contact (inverted coplanar) device architecture. Note that both the contact resistance and the intrinsic channel mobility vary noticeably, both within the same substrate and from one substrate to the next, despite the fact that all substrates were fabricated simultaneously (i.e., placed side-by-side onto the substrate holder for each deposition).
